# Planning for a pandemic: Mitigating risk to radiation therapy service delivery in the COVID‐19 era

**DOI:** 10.1002/jmrs.406

**Published:** 2020-06-22

**Authors:** Nigel Anderson, Kenton Thompson, Judy Andrews, Brent Chesson, Alison Cray, Damien Phillips, Michelle Ryan, Sally Soteriou, Glenn Trainor, Nilgun Touma

**Affiliations:** ^1^ Department of Radiation Therapy Services Peter MacCallum Cancer Centre Melbourne Victoria Australia

**Keywords:** COVID‐19, Coronavirus, Cancer, Radiation Therapy

## Abstract

The novel coronavirus (COVID‐19) has rapidly impacted all of our lives following its escalation to pandemic status on 11 March 2020. Government guidelines and restrictions implemented to mitigate the risk of COVID‐19 community transmission have forced radiation therapy departments to promptly adjust to the significant impact on our ability to deliver best clinical care. The inherent nature of our tri‐partied professions relies heavily on multidisciplinary teamwork and patient–clinician interactions. Teamwork and patient interaction are critical to the role of a radiation therapist. The aim of this paper is to describe the experience of the Peter MacCallum Cancer Centre’s (Peter Mac) radiation therapy services during the preliminary stages of the COVID‐19 pandemic in minimising risk to patients, staff and our clinical service. Four critical areas were identified in developing risk mitigation strategies across our service: (a) Workforce planning, (b) Workforce communication, (c) Patient safety and wellbeing, and (d) Staff safety and wellbeing. Each of these initiatives had a focus on continuum of clinical care, whilst minimising risk of cross infection for our radiation therapy workforce and patients alike. Initiatives included, but were not limited to, establishing COVID‐Eclipse clinical protocols, remote access to local applications, implementation of Microsoft Teams, personal protective equipment (PPE) guidelines and virtual ‘Division of Radiation Oncology’ briefing/updates. The COVID‐19 pandemic has dictated change in conventional radiation therapy practice. It is hoped that by sharing our experiences, the radiation therapy profession will continue to learn, adapt and navigate this period together, to ensure optimal outcomes for ourselves and our patients.

## Graphical abstract

This commentary describes the operational changes made by the Peter MacCallum Cancer Centre's Radiation Therapy Services during the initial stages of the COVID‐19 pandemic. Changes to clinical operations, to ensure optimal patient and staff safety and wellbeing, whilst maintaining a high‐ quality clinical service, are detailed.

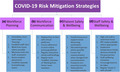

## Background

The novel coronavirus (COVID‐19) has rapidly impacted the livelihoods of many millions of people globally in the opening quarter of 2020. We have witnessed an exponential progression in cases since the first reported case to the World Health Organisation (WHO) in Wuhan, China, on 31 December 2019.^1^ Less than three months later (11 March 2020), we saw the escalation of COVID‐19 to pandemic status. At the time of writing (May 6), the WHO reports in excess of 3.5 million cases globally, including in excess of 240,000 deaths attributed to COVID‐19.^2^ Australia contributes close to 6,800 cases, including 96 deaths, to these figures.^3^

While the world grapples with the unprecedented challenges associated with COVID‐19, health care must adjust to the significant impact this pandemic is having on our ability to deliver best care to our patients. Oncology, and more specifically radiation oncology, is not immune to these challenges. Cancer does not stop in a time where the world, as we know it, has come to a grinding halt. The inherent nature of our tripartite professions, radiation oncology, radiation therapy and medical physics, relies heavily on multidisciplinary teamwork, coupled with an abundance of patient–clinician interaction. Never before has our ability to deliver these exceptional standards of care been compromised on a scale equivalent to what we are currently experiencing. Government guidelines and restrictions with respect to physical and social distancing strike at the very core of our day‐to‐day norm.

The Australian radiation oncology community has no prior experience in dealing with a pandemic. However, we are fortunate to have access to the published experiences of the Singapore Radiation Oncology community during the unprecedented severe acute respiratory syndrome (SARS) outbreak of 2003, to help shape our work practices in readiness for the prolonged nature and peak of the COVID‐19 pandemic.^4^

Whilst many parts of Asia and Europe were not afforded the luxury of a brief preparatory window, we in Australia were given this critical period of time to instigate a number of changes to the delivery of our clinical operations. This not only enabled us to minimise the obvious health‐associated risks to our patients and our staff, but gives us the best opportunity to minimise impact on our ability to provide the continuum of cancer care to those who need it.

The aforementioned pillars of teamwork and patient interaction are paramount to the role of a radiation therapist. The aim of this paper is to describe the experience of the Peter MacCallum Cancer Centre’s (Peter Mac) radiation therapy services (RTS) during the preliminary stages of the COVID‐19 pandemic, through outlining a number of risk mitigation strategies that have been implemented across our service. It is hoped that this document will further compliment the collegiate approach already undertaken by Australian radiation therapy service leaders, in providing a uniform, optimal service, ensuring both patient and staff safety wherever possible. Whilst the strategies discussed may not be applicable to every radiation therapy department, it is hoped that by sharing our experiences as a large, multi‐campus, provider of radiation therapy in Australia, we will further open the conversation amongst our workforce and learn from each other in what are truly unprecedented times for us all. Furthermore, a number of our campuses are aligned to host hospitals, where the complexities of aligning their own protocols (e.g. Personal Protective Equipment (PPE), temperature checks on entry) with Peter Mac’s provide unique and additional challenges. Finally, this publication will reinforce to our patients that they should remain confident that their safety, physical and mental wellbeing remain our priority as we navigate our way through this challenge.

## Risk Mitigation

**Figure 1:**
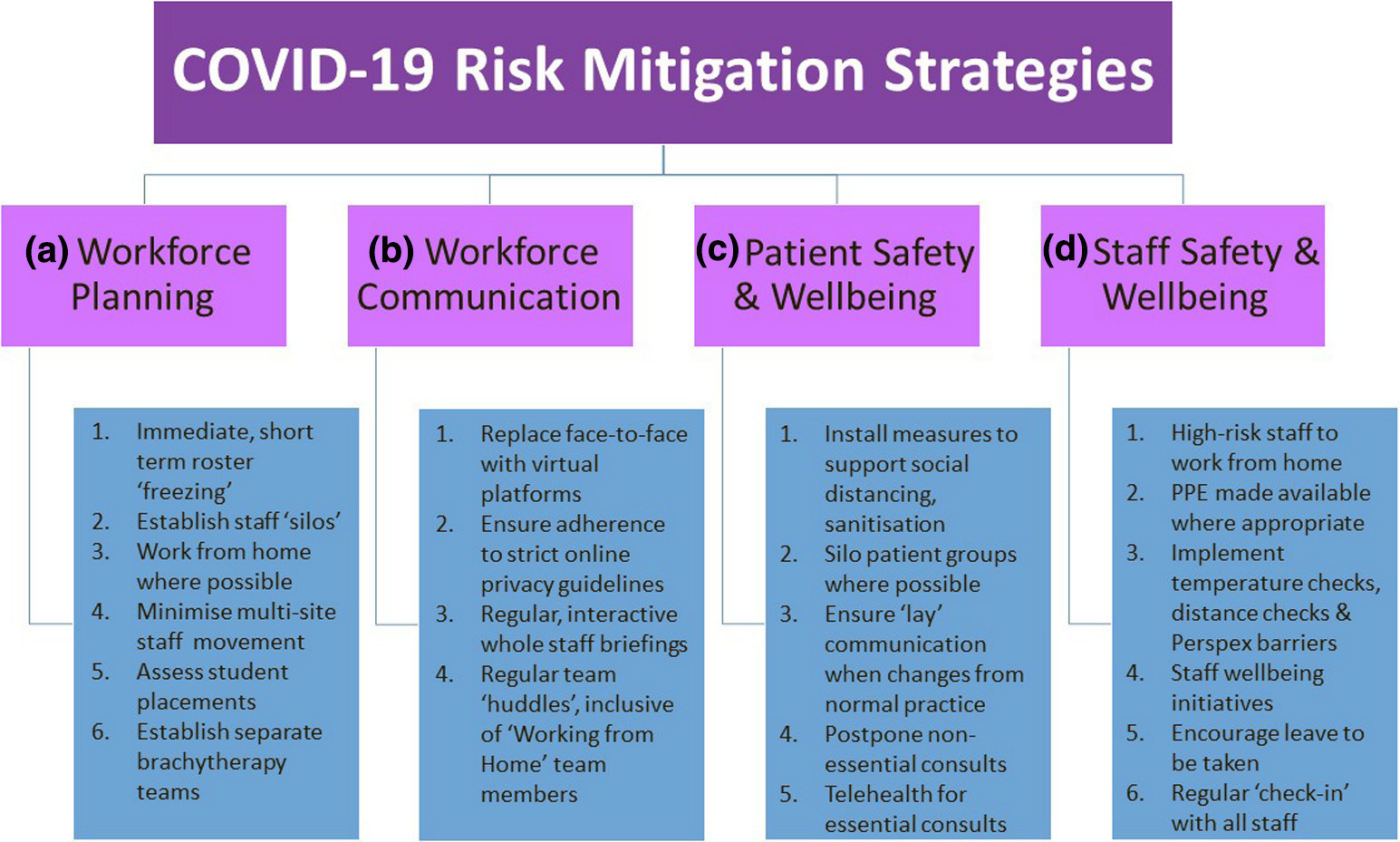
Summary of COVID‐19 risk mitigation strategies initiated by the Peter MacCallum Cancer Centre’s Radiation Therapy Services.

Four critical areas were identified in developing risk mitigation strategies across our service (refer to Figure 1 for summary):

- Workforce planning
- Workforce communication
- Patient safety and wellbeing
- Staff safety and wellbeing

### Workforce planning

As per the rationale behind community social and physical distancing guidelines, Peter Mac’s RTS have implemented a strategy that aims to best align to these recommendations. Cognisant that our ‘normal’ working environment demands interaction, it was imperative to maintain this in some form, whilst remaining mindful that essential changes were required to minimise the operational impact should a staff and/or patient COVID‐19 infection impact our department.

As a consequence of these and many other operational considerations, the following workforce models were adopted. It should be noted that these models are current at the time of writing, and are constantly being reviewed and refined to meet the needs of the rapidly evolving pandemic and associated guidelines given daily in updates from the Department of Health and Human Services (DHHS) in Victoria:

1. Radiation therapy rosters frozen to remove unnecessary staff movement and interaction.
2. Establishment of ‘Patient‐facing’ teams, for each distinct linear accelerator, pre‐planning (computed tomography (CT) simulation) and superficial radiation therapy (SXRT) areas.
3. Establishment of ‘Non‐patient‐facing’ teams, who remain exclusively in the planning room.
4. Establishment of a small additional (non‐patient‐facing) planning team, who undertake planning work but located in a space removed from that of the core planning team. This team do planning work, but remain separate from the planning room so that they can be re‐deployed to patient‐facing areas to backfill as the need arises.
5. Senior RT staff to avoid moving between ‘patient‐facing’ and ‘non‐patient‐facing’ areas, to mitigate risk of cross infection across all staffing levels.
6. Gradual transition of appropriate staff to a Working from Home (WFH) model. Typically, this has been afforded to radiation therapy coordinators with limited operational responsibilities and planning staff with the skills and experience to work autonomously from home.
7. Establishment of two distinctly separates Brachytherapy teams, to mitigate the risk to the service should a staff member(s) become exposed to COVID‐19 and isolation procedures be employed.
8. Movement of all staff between teams at a campus and across sites ceased unless deemed absolutely essential, to minimise the risk of cross infection. This also enables staff to be ‘called in’ should the need arise at any given campus/s.
9. Senior RT meetings traditionally held in person have been virtualised to mitigate risk of losing department leadership due to COVID‐19 exposure.
10. Implementation of rostering that sees 2 distinct treatment teams running a single linac over extended hours of operation that also incorporates a daily component of WFH for each team.
11. Suspension of short (4–6 week) radiation therapy student clinical placements that would require movement between patient‐facing and non‐facing teams.
12. Modification of rosters for employed learners (Interns and final year Monash University Professional Clinical Placements (PCP)) to remain in facing or non‐facing teams.

Additional considerations to support the above workforce models and mitigate the risk of cross infection:

1. Staff should avoid moving between the clinical and non‐clinical zones.
2. Staff unable to take breaks with staff from other teams. This applies to all of the craft groups. Separate tea areas have also been established.
3. Cancellation of non‐essential meetings and projects, and performing virtual meetings where required.
4. Information technology (IT) infrastructure and staff education to support remote access to all resources routinely accessed at work (e.g. Treatment Planning System (TPS), Oncology Information System (OIS), Hospital Electronic Medical Record (EMR), Picture Archiving and Communication System (PACS)).

### Workforce communication

As previously mentioned, the very nature of the role of a radiation therapist demands teamwork and constant communication. The creation of ‘siloed’ teams has significantly impacted our traditional forms of communication, not just within our individual campuses, but also across our campuses. In order to minimise this impact, we have developed/expanded the following platforms to support staff communication:

1. Initially, whilst other platforms were being developed, communication between patient‐facing and non‐facing groups should be by phone wherever practical.
2. Initially, multiple short‐term solutions (Workplace Chat (Facebook, Menlo Park, CA), Slack (Slack Technologies, San Francisco, CA), Zoom (Zoom, San Jose, CA)) were implemented to fill any communication void. Various teams opted for the solution best suited to their immediate needs, whilst remaining cognisant of the privacy limitations of each platform, and instituting strict guidelines to respect this in the clinical setting. Staff should not transcribe/write any patient identifiers in any of these platforms. All verbal communication/screen sharing incorporating patient identifiers is restricted to the secure, in‐house Peter MacCallum Cancer Centre Video/Teleconference platform or phone call.
3. Our institution‐wide rollout of Microsoft Office 365 and Microsoft Teams (Microsoft, Redmond, WA) was brought forward, to provide a uniform platform for communication to the wider RTS team, together with smaller teams (e.g. campus planning clusters, education teams, senior RT teams).
4. All essential meetings moved to either the internal Peter Mac video conferencing platform or Microsoft Teams.
5. Bi‐weekly ‘Division of Radiation Oncology’ YouTube (YouTube, San Bruno, CA) briefing/update (featuring the Clinical Director and RTS Director), with capability for staff questions via an online, interactive platform. A daily organisation‐wide equivalent briefing has also been initiated.
6. Bi‐daily huddles to aid communication with WFH team.

Additional considerations to support the above workforce communication strategies:

1. When operating on non‐traditional, multimedia platforms, to be cognisant not to include patient identifiers
2. Many video conferencing platforms support recording. Consider recording interactions so that those unable to attend are able to view afterwards (e.g. a YouTube demonstration was undertaken to detail the new electronic patient consent process – see ‘Patient Safety and Wellbeing, Step 9’ below)

### Patient safety and wellbeing

Whilst we have had to rapidly adapt to the changing landscape and our ability to deliver world’s best cancer care, we must be incredibly mindful of our patients during this incredibly challenging time, even without the added stressors of the COVID‐19 pandemic. Furthermore, a large majority of our patients fall into the COVID‐19 higher risk groups described by our health authorities. Therefore, it is critical that we modify their experience to mitigate their risk of COVID‐19 infection, whilst limiting the impact on their radiation therapy. Peter Mac has implemented the following measures to mitigate these risks:

1. Removal of some furniture in the patient waiting room to support social distancing between patients. We have also removed magazines, books, puzzles etc.
2. Ask patients to ideally attend their appointment alone, or limit to one carer where needed.
3. Where possible, split the patient group into ‘morning’ and ‘afternoon’ groups, to establish an equivalent silo in patients to that of staff to minimise the risk of cross‐infection.
4. Ask patients when they are contacted with their pre‐planning appointment if they have travelled overseas, had contact with a positive case or have any of the presenting symptoms. If they have travelled/had contact and are symptomatic we would ask them to seek medical advice outside of Peter Mac before attending. These questions will also be asked the day prior to the 1^st^ Fraction, where patients also have an opportunity to ask any questions in advance of their visit to allay fears and concerns about the treatment and/or COVID‐19. The same protocol is applied to patient clinic and treatment review appointments.
5. Provide patients with appropriate, ‘lay’ communication when there is a change of practice that is introduction of PPE.
6. Established procedures (campus specific) that detail processes that if/when we are required to treat a COVID‐19 positive patient, how we will transition the patient through the department for radiation therapy, whilst mitigating contact points with staff and other patients, for example, staff in appropriate PPE meet patient at car, bring in for radiation therapy in a wheelchair, then taken straight back to car post‐treatment).
7. Patients are asked to use hand sanitiser before entering the bunkers and again upon exiting. Additionally, Peter Mac has transitioned to Clinell (GAMA Healthcare, UK) from Tuffie (Vernacare, UK) Wipes due to its demonstrated effectiveness against COVID‐19^5^.
8. Where possible, routine consultations are postponed, cancelled or moved to telehealth.
9. Where initial radiation oncologist consult has been undertaken via telehealth, an electronic patient consent process has been developed through our MOSAIQ (Elekta, Stockholm, SE) OIS to enable all consent requirements to be met.
10. Trial being performed at our Peter Mac Sunshine campus to perform all pre‐planning and first day treatment information/education sessions the day before via phone. Early indications are encouraging, with a significant reduction in staff/ patient face‐to‐face interactions.
11. Eclipse (Varian Medical Systems, Palo Alto, CA) Clinical Protocols created for hypo‐fractionation prescribed where clinically appropriate to reduce number of patient visits.

### Staff safety and wellbeing

The safety and wellbeing of radiation therapists are of paramount importance during these challenging times. We cannot comply entirely to social distancing guidelines due to the inherent nature of our role. Therefore, it is critical that measures are put in place to minimise the risk of cross infection and exposure to COVID‐19, for not only health and wellbeing of the radiation therapist, but of their loved ones, and the continuity of clinical operations across our organisation. Furthermore, the impact of the pandemic poses challenges physically, emotionally and mentally we have never faced before – both inside and outside the workplace. We must therefore consider these ramifications and provide supports to our teams wherever possible. Peter Mac has initiated the following measures to maximise staff safety and wellbeing during the pandemic, and possibly, beyond:

1. Staff with pre‐existing medical conditions or those that fall into high‐risk categories have been encouraged to WFH wherever possible. Other high‐risk situations are also considered in the WFH model.
2. Personal protective equipment (PPE) has been made available for staff in CT simulation and treatment for two (2) specific scenarios:
  - CT simulation and treatment of head and neck patients in thermoplastic cast.
  - CT simulation and treatment of patients with thoracic malignancy.
    For these two scenarios, we recognise that the patient is at an increased risk of coughing during the radiation therapy procedure, and is limited in their ability to exercise social distancing and cough etiquette. For these procedures, we will therefore use PPE consisting of goggles and a mask only. This applies to all patients of the above two categories where a patient does not have confirmed or suspected COVID‐19 (i.e. patients with confirmed or suspected COVID‐19 must adhere to local hospital‐wide protocols).
3. PPE made available when deemed clinically appropriate, and in alignment with local and government PPE directives.
4. Installation of perspex screens and distance indicators at reception desks to support spatial distancing recommendations coupled with temperature checks and hand sanitiser stations upon hospital entry.
5. Amendment to our paediatric general anaesthesia workflows is required as this is an aerosol‐generating procedure. Full PPE is required for treating staff and the anaesthetics team (mask, goggles, gown and gloves). Social distancing between the radiation therapists and anaesthetists has been achieved with support from biomedical engineers to develop a solution for remote monitoring of anaesthetics equipment in a room adjacent to the control room. Scheduling allows for an interval between successive GA cases to provide additional cleaning and air clearance in the bunker for higher risk cases that is where the patient is at elevated risk of COVID‐19 or is actually COVID‐19 positive.
6. The Compassion and Resilience Education (CARE) programme has been launched amongst RTS, to develop a number of ‘CARE Champions’ across our division. A CARE Champion is a role model in their work area when it comes to discussing and advocating good mental health practices. They also act as peer support and are a point of contact for anyone looking for a listening ear, support and/or referrals for care. They are not expected to be a counsellor for mental health and instead, act as a point of contact and referral to direct a colleague to get the support they need. Local training is provided.
7. The initiation of hospital wellbeing initiatives, including a hospital intranet page dedicated to COVID‐19, mindfulness meditation videos techniques and tips on home isolation.
8. Integration of department supported ‘socially distanced’ morning teas for siloed/WFH staff and virtual ‘after work drinks’ are two of a number of initiatives that have been undertaken in an attempt to boost staff morale.

Additional considerations to support staff safety and wellbeing:

1. As the pandemic situation elongates, online CPD resources are being developed, to counter the void in traditional CPD options (e.g. conferences, local in‐services etc). They are by no means mandatory, but provide a resource for staff who are keen to engage.
2. Encouraging leave to continue to be taken (where possible), to ensure a mental break.
3. Regular ‘check‐ins’ with staff who are WFH, to ensure their needs are being met, they are well and safe, and any barriers to their work are being addressed.
4. RTS wide survey circulated to gain feedback on current working arrangements and communication strategies, enabling staff input to workforce planning moving forward.

## Conclusion

The first few months of 2020 has thrown us challenges that most, if not all of us, have never faced before, and will hopefully never have to face again in our lifetimes. The ability for radiation therapists, as part of the wider radiation oncology multidisciplinary team, to deliver ‘business as usual’ has been greatly affected.

Whilst extremely challenging, this pandemic has been met with exceptional teamwork, agility and understanding that belies the way we approach our work. It has necessitated change in a number of our practices, many of which we and our patients will continue to reap the benefits of long after COVID‐19 has passed through our radiation therapy departments.

We see this as a unique opportunity at Peter Mac RTS to share our experiences during this very different time. It is by no means the only way to approach this unprecedented challenge. Many of you will have taken a different approach based on your local needs. It has truly challenged the way we think, operate and communicate in so many different ways. We hope that by us sharing our experiences, we will further open the discussion and encourage all radiation therapy departments to share their experiences, nuances and strategies to best tackle the challenges this pandemic has forced upon us.

It is quite the cliché – one we have heard many times over the preceding months – but we really are ‘all in this together’. The more we can help each other by being open and transparent, the more likely we are to be the beneficiaries of optimal outcomes for ourselves and our departments, and ultimately, our patients.

Stay safe everyone!
